# Levobupivacaine inhibits proliferation and promotes apoptosis of breast cancer cells by suppressing the PI3K/Akt/mTOR signalling pathway

**DOI:** 10.1186/s13104-020-05191-2

**Published:** 2020-08-17

**Authors:** Akosua Kotaa Kwakye, Sylvanus Kampo, Jiaxin Lv, Muhammad Noman Ramzan, Seidu A. Richard, Aglais Arredondo Falagán, Jerry Agudogo, Evans Atito-Narh, Qiu Yan, Qing-Ping Wen

**Affiliations:** 1 Department of Anaesthesiology, Dalian Medical University, Dalian, China; 2 Department of Anaesthesiology, First Affiliated Hospital of Dalian Medical University, Dalian, China; 3 Department of Biochemistry and Molecular Biology, Dalian Medical University, Dalian, China; 4 Department of Anaesthesia and Critical Care, School of Medicine, University of Health and Allied Sciences, Ho, Ghana; 5 Department of Biochemistry and Molecular Medicine, School of Medicine and Health Sciences, University for Development Studies, Tamale, Ghana; 6 Departments of Anaesthesia and Critical Care, Ridge Hospital, Accra, Ghana; 7 Department of Medicine, Princefied University, Ho, Ghana

**Keywords:** Levobupivacaine, Proliferation, Invasion, Apoptosis, Breast cancer

## Abstract

**Objective:**

This study aimed to test the hypothesis that levobupivacaine has anti-tumour effects on breast cancer cells.

**Results:**

Colony formation and transwell assay were used to determine breast cancer cells proliferation. Flow Cytometry (annexin V and PI staining) was used to investigate breast cancer cells apoptosis. The effects of levobupivacaine on cellular signalling and molecular response were studied with Quantitative Polymerase Chain Reaction and western blot. Induction of apoptosis was confirmed by cell viability, morphological changes showed cell shrinkage, rounding, and detachments from plates. The results of the western blot and Quantitative Polymerase Chain Reaction indicated activation of active caspase-3 and inhibition of FOXO1. The results of the flow Cytometry confirmed that levobupivacaine inhibited breast cancer cell proliferation and enhanced apoptosis of breast cancer cells. Quantitative Polymerase Chain Reaction and Western blot analysis showed increased p21 and decreased cyclin D. Quantitative Polymerase Chain Reaction and western blot analysis showed that levobupivacaine significantly increased Bax expression, accompanied by a significant decreased Bcl-2 expression and inhibition of PI3K/Akt/mTOR signalling pathway. These findings suggested that levobupivacaine inhibits proliferation and promotes breast cancer cells apoptosis in vitro.

## Introduction

Breast cancer is one of the most recorded cancer illness among women [1,2]. In the United States, it is estimated that more than 40,000 women die every year from breast cancer-related illness, despite the advance in chemotherapy and targeted treatments [3].

Molecular signalling pathways that are involved in breast cancer transformation have become targets for treatment [4]. The mechanisms of the PI3K/Akt/mTOR signalling pathway have present some promising targets for cancer treatments. This signalling pathway hinders the functions of several tumour suppressor genes such as Bad, GSK3, FOXO transcription factors, and tuberin/hamartin complex which control cell survival, proliferation, and growth [5–10]. Suppressing this signalling pathway may inhibit cancer cells proliferation and also stimulate them toward cell death.

The growing evidence of local anaesthetics inhibiting cancer cell growth seems promising, though limited [11]. At the tissue level, administration of a certain amount of local anaesthetics topical or local has shown to have a direct inhibitory effect on the action of epidermal growth factor receptor (EGFR) which is a potential target for anti-proliferation in cancer cells [12,13]. Evidence also shows that ropivacaine and lidocaine hinder cancer cells growth, invasion, migration and enhance apoptosis of lung cancer cells [14–17]. To the best of our knowledge, the effect of levobupivacaine on breast cancer cells is yet to be determined. The present study, therefore, aimed to investigate the anti-tumour effects of levobupivacaine on breast cancer cells.

## Main text

### Materials and methods

#### Ethics statement

The ethical committee of the Dalian Medical University First Affiliated Hospital approved for this study to be carried out.

#### Cell culture

We purchased MCF-7 and MDA-MB231 breast cancer cells from the ATCC (Beijing Zhongyuan limited, China). We maintained the MCF-7 and MDA-MB-231 cells with high-glucose DMEM or DMEM/F12 (Gibco, USA) medium. The medium was supplemented with 10% fetal bovine serum (FBS) (Gibco, USA), penicillin 100 units/ml and streptomycin 100 µg/ml (TransGen Biotech, China) to maintain the cells. The MCF-7 and MDA-MB231 cells were then maintained in an incubator at 37 ^º^C humidified air with 5% CO_2_ atmospheric condition. The cells were routinely subcultured subsequently.

#### Antibodies and reagents

#EPR17671 Akt monoclonal Antibody (Abcam, China), #Y391 mTOR Polyclonal Antibody (Abcam, China) #A2845 Bcl-2 Polyclonal Antibody (ABclonal Technology), #A11550 Bax Polyclonal Antibody (ABclonal Technology), #A0265 PIK3CA Polyclonal Antibody (ABclonal Technology), #A2934 FOXO1 Polyclonal Antibody (ABclonal Technology), #EPR21032 Active caspase 3 monoclonal Antibody (Abcam, China), #AFO931 Cyclin D1 Polyclonal Antibody (Affbiotech, China), #AF6290 p21 Polyclonal Antibody (Affbiotech, China), Anti-mTOR (phospho S2448) Antibody (Abcam, China), #PA5-17,387 Phospho-PI3K p85/p55 (Tyr458, Tyr199) Polyclonal Antibody (ThemoFisher Scientific), Pospho-pan-AKT1/2/3 (Ser473) Antibody (Affbiotech, China), Peroxidase-conjugated goat anti-rabbit IgG (Proteintech, China); PRAP antibodies (Proteintech, China), and GAPDH antibodies (Proteintech, China).

#### Cell viability assay and IC50

We determined the MCF-7 and MDA-MB 231 cells viability using CCK-8 assay. Levobupivacaine at a concentration of 0, 1, 2 or 3 mM was used to treat MCF-7 and MDA-MB 231 cells plated in 96-well plates (1 × 10^4^ cells/well) and then incubated for 12, 24, or 48 h respectively in an incubator at the atmospheric condition of 37 °C with 5% CO_2_. The rest of the procedures used for the CCK-8 assay were the same as described elsewhere [18].

#### Flow cytometry

Annexin V and propidium iodide (PI) staining assay were used to investigate the apoptosis of MCF-7 and MDA-MB 231 cells following levobupivacaine treatment. After treating the cells for 24 h, 0.25% trypsin was used to harvest the treated cells and centrifugation at 1400 rcf for 10 min. The MCF-7 and MDA-MB 231 treated cells were again suspended with 1 × Binding Buffer, and then 5 µl of fluorochrome-conjugated annexin V (Sigma-Aldrich, Saint Louis, USA) was added into 100 µl of the cell suspension to stain intracellular phosphatidylserine (PS). The cells were then incubation in a dark under room temperature. The cells were again suspended and 5 µl propidium iodide staining solution (Sigma-Aldrich, Saint Louis, USA) added into 100 µl of the cell suspension. We detected the percentage of the apoptotic cells via FlowJo software (Treestar, Ashland, USA) and Flow cytometry (FACS Calibur, Becton Dickinson, and Sunnyvale, CA, USA).

#### Quantitative polymerase chain reaction (qPCR)

The procedures used for the qPCR were the same as previously described [18]. The primers sequences were: BAX: 5-TGGCAGCTGACATGTTTTCTG-3 (F), 5-TCCCGGAGGAAGTCCAATG-3 (R). BCL_2_ 5-ACGGTGGTGGAGGAGCTCTT-3 (F), 5-GCCGGTTCAGGTACTCAGTCAT-3 (R). p21 5-GCGACTGTGATGCGCTAATG-3 (F), 5-GAAGGTAGAGCTTGGGCAGG-3 (R). GAPDH: 5′-CATGTTCGTCATGGGTGTGAA-′3 (F), 5′-GGCATGGACTGTGGTCATGAG-3′ (R).

#### Western blot

At the log phase of treated MCF-7 and MDA-MB 231 cells growth, we harvested the cells and then washed twice with ice-cold PBS. The rest of the procedures used for the western blot were the same as described elsewhere [18].

#### Colony formation assay

The procedures used for the colony formation assay were the same as previously described [18].

#### Transwell assay

The MCF-7 and MDA-MBA-231 cells (5 × 10^4^) that were pre-treated with different dose of Levobupivacaine (0, 1, 2 mM) for 24 h and resuspended in culture medium with the same concentrations of levobupivacaine were seeded onto the coated membrane in the upper chamber of the transwell (24-well millicell cell culture insert, 12 mm diameter, 8 μm pores; Merck KGaA, #P18P01250, China). The procedures used for the Transwell assay were the same as previously describe [18].

#### Data analysis

Values were expressed as the mean ± SD. Statistical analysis was performed with GraphPad Prism version 5.01(GraphPad Software, La Jolla, CA, US). One-way ANOVA was used to measure significance (*p* < 0.05). Dunnett’s post hoc tests were used to test the difference between groups.

## Results

### Levobupivacaine decreases breast cancer cell invasion

Transwell assay analysis showed significantly decreased in the invasion ability of MCF-7 and MDA-MB-231 cells in a dose-dependent manner compared with the untreated cells (Additional file 1: Fig. S1a, b). 

### Levobupivacaine inhibits proliferation in breast cancer cells

**Fig. 1:**
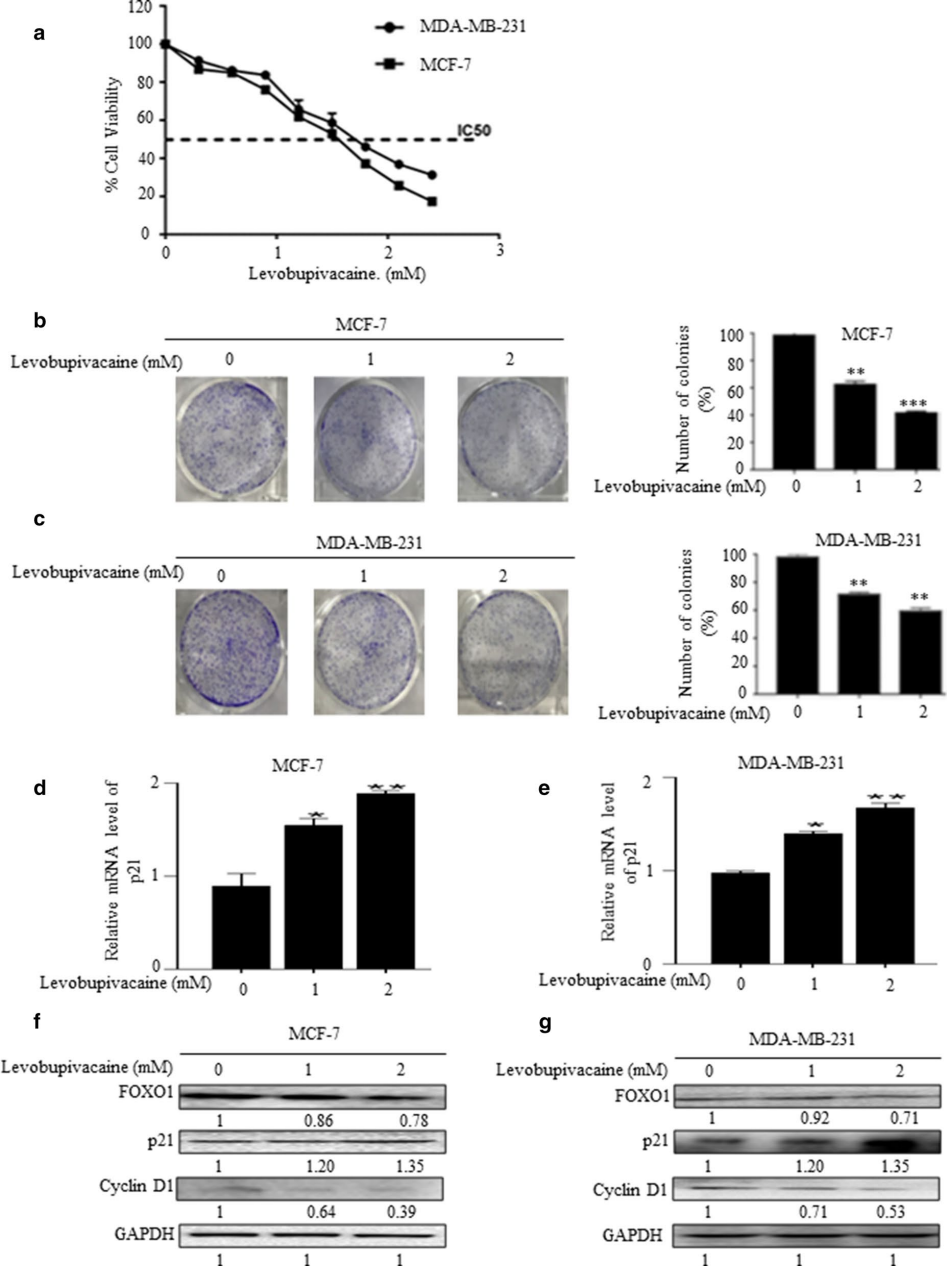
Levobupivacaine inhibits proliferation in breast cancer cells. MCF-7 and MDA-MB-231 cells were treated with different concentrations of levobupivacaine. **a** Cell viability was measured by CCK-8 assay**.** IC50 results of levobupivacaine on MCF-7 and MDA-MB 231 cells. **b**, **c** Colony formation of MCF-7 and MDA-MB 231 cells treated with various concentrations of Levobupivacaine and stained with crystal violet. **d**, **e** The mRNA expression levels of p21 and GAPDH were analysed by qPCR. **f**, **g** Protein expression assessment of MCF-7 and MDA-MB-231 cells by western blot against antibodies FOXO1, p21, Cyclin D1 and GAPDH used as control**.** The data was statistically significant at * indicates *P *< 0.05; ** indicates *P *< 0.01; *** indicates *P *< 0.001 compared with untreated cells. This data corresponds to the mean ± SEM of three independent experiments

The MCF-7 and MDA-MBA-231 cell viability decreased as the concentrations of levobupivacaine (0, 1, 2 or 3 mM) increased. The MCF-7 cells showed a 50% cytotoxic effect, while the MDA-MB-231 cells showed a similar cytotoxic effect of 40% (Fig. 1a). Under a fluorescence microscope, cells treated with levobupivacaine showed morphological changes including cell rounding, cell shrinkage, and cells detachment from the plates (Additional file 2: Fig. S2a, b). The viability of breast cancer cells decreased in a dose-dependent manner. The results showed significantly decreased in the number of clones of the treated cells compared with the untreated cells (Fig. 1b, c). The data showed that the mRNA level of p21 significantly increased following levobupivacaine treatment (Fig. 1d, e). Western blot analysis showed a similar increased in p21 and decreased in FOXO1 and cyclin D1 expressions in a dose-dependent manner compared with the untreated cells (Fig. 1f, g; Additional file 3f, g).

### Levobupivacaine promote apoptosis in breast cancer cells

**Fig. 2:**
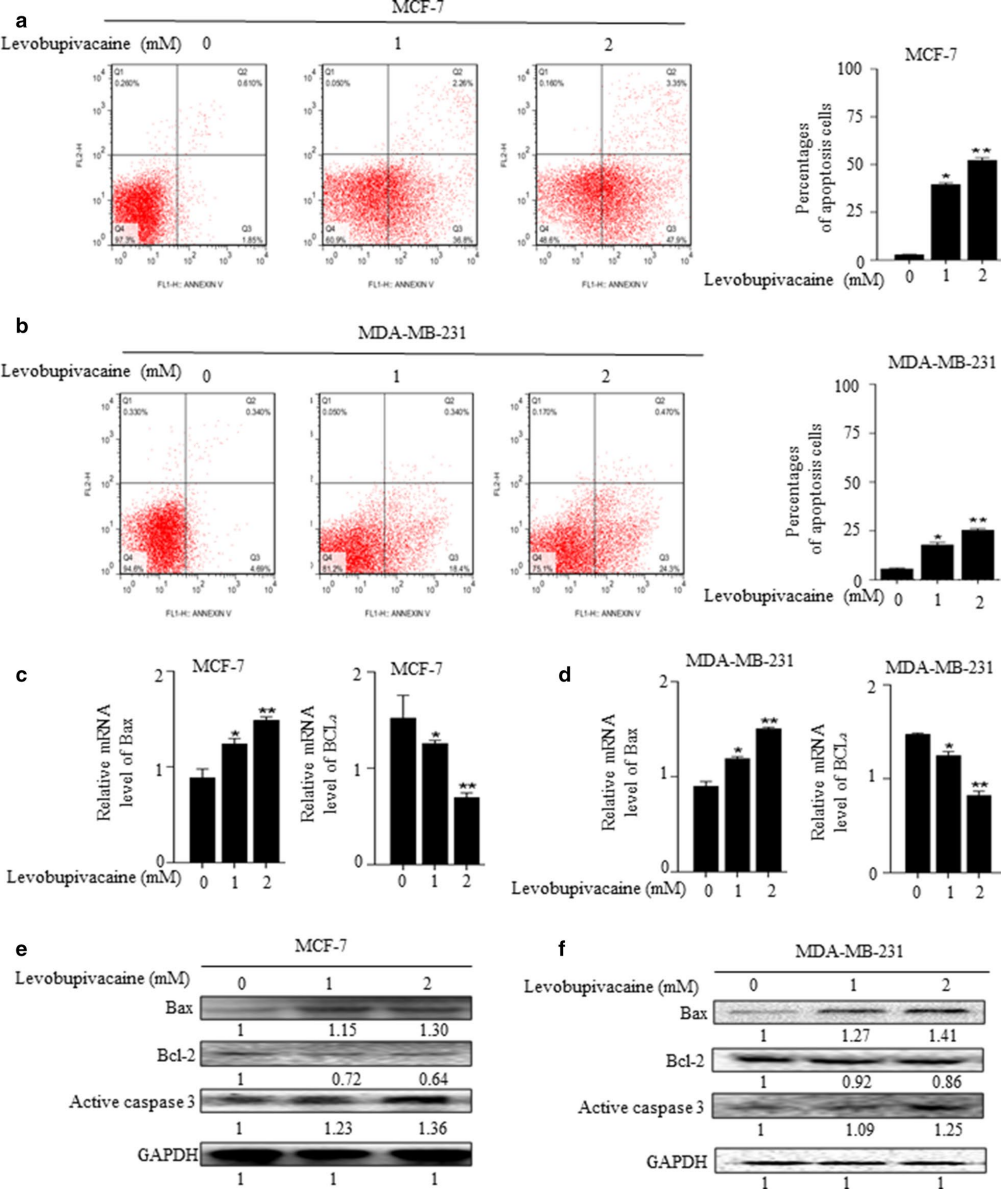
Effects of levobupivacaine on apoptosis of breast cancer cells. **a**, **b** MCF-7 and MDA-MB 231 cells were treated with different concentrations of levobupivacaine for 24 h. The cells were then stained with fluorescein-conjugated annexin V and PI and analysed by flow cytometry. Error bars represent standard error of the mean. *P* < 0.05 versus the control. **c**, **d** Relative gene expression of Bax and Bcl-2 following the treatment of breast cancer cells with different concentrations of levobupivacaine for 24 h and analysed by qPCR. **e**, **f** MCF-7 and MDA-MB 231 cells were treated with different concentrations of levobupivacaine for 24 h and the activities of Bax, Bcl-2, and Active caspase 3 were examined by Western blot analysis using specific antibodies. GAPDH was used as internal controls. The data was statistically significant at * indicates *P *< 0.05; ** indicates *P <* 0.01 compared with control. The data correspond to the mean ± SEM of three independent experiments

Levobupivacaine significantly reduced the number of cells showing nuclear staining when compared with the untreated cells (Fig. 2a, b). The qPCR data showed a decreased in Bcl-2 and increased in Bax expressions in MCF-7 and MDA-MB-231 cells compared with the untreated cells (Fig. 2c, d). Western blot analysis also showed a similar decreased in Bcl-2 and increased expressions of active caspase 3 and Bax compared with the untreated cells (Fig. 2e, f; Additional file 3e, f).

### Levobupivacaine inhibits proliferation and promotes apoptosis in breast cancer through PI3K/Akt/mTOR signalling pathway

**Fig. 3:**
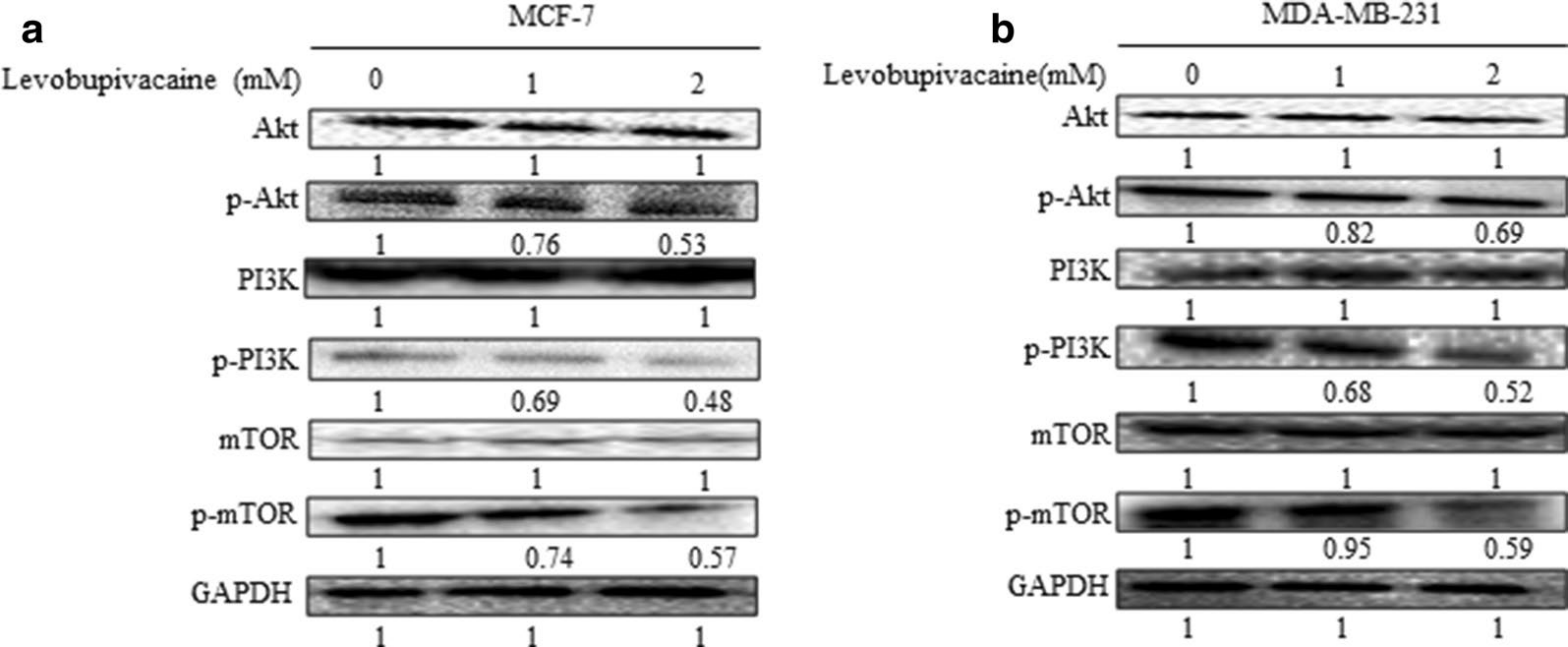
MCF-7 and MDA-MB 231 cells were treated with different concentrations of levobupivacaine for 24 h. **a**, **b** The cells were lysed and subjected to 12% SDS-PAGE and analysed by western blotting and probed with specific antibodies p-PI3K, p-Akt, and p-mTOR. The results showed a decrease in the expressions of p-PI3K, p-Akt, and p-mTOR proteins. GAPDH was used as internal controls. The data represent the mean ± SD of three independent experiments

Western blot analysis showed a significant decreased in the expression of the nuclear localization of p-PI3K, p-Akt, and p-mTOR compared with the untreated cells (Fig. 3a, b; Additional file 3a, b).

## Discussion

Breast cancer remains a common cause of mortality among women worldwide. Though current orthodox drugs have demonstrated promise in breast cancer therapy, its treatment options remain limited. These, therefore, supports the concept that effective therapeutic approaches for breast cancer are critically needed. Several retrospective studies have demonstrated that regional anaesthesia is associated with a decreased risk of recurrence or metastasis of multiple carcinomas, including breast, prostate and cervical cancers [19–21]. Recent growing evidence demonstrates that local anaesthetics have an anti-tumour effect and may suppress the motility of cellular function and invasiveness more likely via voltage-gated sodium channel inhibition [19,20]. A study report indicates that lidocaine inhibits the growth of human hepatocellular carcinoma cells (HCC) by increasing the Caspase 3 activity, whereas ropivacaine inhibits the growth of HCC cells by stopping the cell cycle in G2 phase [21]. Lee et al. demonstrated that local anaesthetics potentiate TNF-α mediated apoptosis in HK-2 cells [22]. The cellular modification of treated cells is likely dependent on the duration of exposure and the dose of the local anaesthetic [22–36]. In this study, we employed MCF- 7 and MDA-MB-231 cells as models and found that different concentrations of levobupivacaine could effectively inhibit breast cancer cell proliferation and promote apoptosis in vitro. The anti-proliferation and apoptosis effects observed in this study suggest that levobupivacaine may have therapeutic effects on breast cancer.

PI3K/Akt/mTOR signalling pathway plays a vital role in cell proliferation, survival, development, metabolism, motility and regulation of the immune response. Breast cancer cell resistance to therapies can result from the activation of PI3K/Akt/mTOR signalling pathway [37–40]. This has made the PI3K/Akt/mTOR signalling pathway an important object of study for understanding the development and progression of breast cancer. In patients with breast cancer, PI3K/Akt/mTOR signalling pathway can be a target for diagnostic, prognostic and treatment purposes [2,41–46]. Akt plays a role in the activation and inactivation of many transcription factors. Activation of Akt correlated with the activation of mTOR. Phosphorylation of the FOXO proteins by Akt may results in cytoplasmic retention by interacting with other proteins, thereby isolating them from their targeted genes. Cyclin D1 classified as a pro oncogene is often over-expressed in several human malignancies including breast, colon, lung and prostate cancers [40,42]. Reports show that over-expression of cyclin D1 and under-expression of tumour suppressor p21 is required for cancer initiation as it is confirmed that down-regulation of cyclin D1 and over-expression of p21 in xenograft model discontinues the formation of cancer in the early stages [43]. Datta et al. reported that Akt can phosphorylate the pro-apoptotic Bcl-2 family member Bad causing its isolation from the mitochondrial membrane by other proteins [44]. Local anaesthetics modify the protein levels of key members of the Bcl-2 family in a manner that presents an increase in the ratio of Bax/Bcl-2, which may contribute to the response of cancer cells to apoptosis. In the present study, the role of levobupivacaine on the expression of PI3K, Akt, and mTOR was investigated to illustrate the potential molecular mechanism. We observed a significantly decreased expression of p-Akt, p-PI3K, p-mTOR and subsequent decreased expression of FOXO, Cyclin D1 and Bcl-2 following levobupivacaine treatment which correlated with decreased breast cancer cells proliferation and increased apoptosis. These emerging pieces of evidence suggest that levobupivacaine may inhibit proliferation and promote apoptosis by suppressing PI3K/Akt/mTOR signalling pathway, which demonstrated an anti-tumour effect on breast cancer cells in this study.

## Conclusion

levobupivacaine has the potency of reducing breast cancer cell viability, proliferation and also causes cell death by suppressing the PI3K/Akt/mTOR signalling pathway. These findings could lead to clinical studies which will seek to examine the anti-cancer effects of levobupivacaine and may also increase the benefits in cancer patient as well as improve patient care.

## Limitations

Numerous studies have reported on the anti-tumour effects of local anaesthetics on various cancer cells [46–48]. However, our work is not without limitations. In vivo and clinical studies on the anti-tumour effects of levobupivacaine are needed.

## Supplementary information

**Additional file 1: Figure S1** Levobupivacaine decreases breast cancer cell invasion.

**Additional file 2: Figure S2** Effect of levobupivacaine on the morphology of MCF-7 and MDA-MB 231 cells.

**Additional file 3.** Original gels/blots scan used in Fig. 1f, g; Fig. 2e, f and Fig. 3a, b for MCF-7 and MDA-MB-231 cells.

## Data Availability

The data used and/or analysed in this study are available from the corresponding author upon reasonable request.

## Abbreviations

EGFR: Epidermal growth factor receptor
HCC: Hepatocellular carcinoma cells
NC: Nitrocellulose
PI: Propidium iodide
PS: Phosphatidylserine
qPCR: quantitativepolymerase chain reaction

## References

[1] American Cancer Society (2017). Breast Cancer Facts and Figures 2017–2018.

[2] American Cancer Society (2018). Cancer Facts and Figures 2018.

[3] Siegel R, Naishadham D, Jemal A (2013). Cancer statistics, 2013. CA Cancer J Clin.

[4] Chang YC, Hsu YC, Liu CL, Huang SY, Hu MC, Cheng SP (2014). Local anaesthetics induce apoptosis in human thyroid cancer cells through the mitogen-activated protein kinase pathway. PLoS ONE.

[5] Gomez-Gutierrez JG, Souza V, Hao HY, de Montes Oca-Luna R, Dong YB, Zhou HS, McMasters KM (2006). Adenovirus-mediated gene transfer of FKHRL1 triple mutant efficiently induces apoptosis in melanoma cells. Cancer Biol Ther.

[6] Sunters A, de Fernández Mattos S, Stahl M, Brosens JJ, Zoumpoulidou G, Saunders CA, Coffer PJ, Medema RH, Coombes RC, Lam EW (2003). FoxO3a transcriptional regulation of Bim controls apoptosis in paclitaxel-treated breast cancer cell lines. J Biol Chem.

[7] Fu Z, Tindall DJ (2008). FOXOs, cancer and regulation of apoptosis. Oncogene.

[8] Barnes DM, Gillett CE (1998). Cyclin D1 in breast cancer. Breast Cancer Res Treat.

[9] Sherr CJ, Roberts JM (1999). CDK inhibitors: positive and negative regulators of G1-phase progression. Genes Dev.

[10] Pelengaris S, Khan M, Evan G (2002). c-MYC more than just a matter of life and death. Nat Rev Cancer.

[11] Di Padova  M, Barbieri R, Fanciulli M, Arcuri E, Florida A (1998). Effect of local anaesthetic ropivacaine on the energy metabolism of Ehrlich ascites tumour cells. Oncol Res.

[12] Xing W, Chen DT, Pan JH, Chen YH, Yan Y, Li Q, Xue RF, Yuan YF, Zeng WA (2017). lidocaine induces apoptosis and suppresses tumour growth in human hepatocellular carcinoma cells in vitro and in a xenograft model in vivo. Anesthesiology.

[13] Drasner K (1997). Lidocaine spinal anaesthesia: a vanishing therapeutic index?. Anesthesiology.

[14] Wang HW, Wang LY, Jiang L, Tian SM, Zhong TD, Fang XM (2016). Amide-linked local anaesthetics induce apoptosis in human non-small-cell lung cancer. J Thorac Dis.

[15] Piegeler T, Votta-Velis EG, Liu G, Place AT, Schwartz DE, Beck-Schimmer B, Minshall RD, Borgeat A (2012). Anti-metastatic potential of amide-linked local anaesthetics: inhibition of lung adenocarcinoma cell migration and inflammatory Src signalling independent of sodium channel blockade. Anesthesiology.

[16] Lirk P, Berger R, Hollmann MW, Feigl H (2012). Lidocaine time and dose-dependently demethylates deoxyribonucleic acid in breast cancer cell lines in vitro. Br J Anaesth.

[17] Villar-Garea A, Fraga MF, Espada J, Esteller M (2003). Procaine is a DNA-demethylating agent with growth-inhibitory effects in human cancer cells. Cancer Res.

[18] Kampo S, Ahmmed B, Zhou T, Owusu L, Anabah TW, Doudou NR, Kuugbee ED, Cui Y, Lu Z, Yan Q, Wen Q-P (2019). Scorpion venom analgesic peptide, BmK AGAP inhibits stemness, and epithelial-mesenchymal transition by down-regulating PTX3 in breast cancer. Front Oncol.

[19] Hirata M, Sakaguchi M, Mochida C, Sotozono C, Kageyama K, Yoshihiro K, Munetaka H (2004). Lidocaine inhibits the tyrosine kinase activity of the epidermal growth factor receptor and suppresses proliferation of corneal epithelial cells. Anesthesiology.

[20] Grouselle M, Tueux O, Dabadie P, Georgescaud D, Mazat JP (1990). Effect of local anaesthetics on mitochondrial membrane potential in living cells. Biochem J.

[21] Fraser SP, Diss JKJ, Chioni AM, Mycielska ME, Pan H, Yamaci RF, Pani F, Siwy Z, Krasowska M, Grzywna Z, Brackenbury WJ, Theodorou D, Koyuturk M, Kaya H, Battaloglu E, De Tamburo Bella M, Slade MJ, Tolhurst R, Palmieri C, Jiang J, Latchman DS, Coombes RC, Djamgoz MBA (2005). Voltage-gated sodium channel expression and potentiation of human breast cancer metastasis. Clin Cancer Res.

[22] Le Gac G, Angenard G, Clement B, Laviolle B, Coulouarn C, Beloeil H (2017). Local anaesthetics inhibit the growth of human hepatocellular carcinoma cells. Anesth Analg.

[23] Lee HT, Xu H, Siegel CD, Krichevsky IE (2003). Local anaesthetics induce human renal cell apoptosis. Am J Nephrol.

[24] Unami A, Shinohara Y, Ichikawa T, Baba Y (2003). Biochemical and microarray analyses of bupivacaine-induced apoptosis. J Toxicol Sci.

[25] Villar-Garea A, Fraga MF, Espada J, Esteller M (2003). Procaine is a DNA-demethylating agent with growth-inhibitory effects in human cancer cells. Cancer Res.

[26] Sakaguchi M, Kuroda Y, Hirose M (2006). The antiproliferative effect of lidocaine on human tongue cancer cells with inhibition of the activity of epidermal growth factor receptor. Anesth Analg.

[27] Chang YC, Liu CL, Chen MJ, Hsu YW, Chen SN, Lin CH, Chen CM, Yang FM, Hu MC (2014). Local anaesthetics induced apoptosis in human breast tumour cells. Anesth Analg.

[28] Kawasaki C, Kawasaki T, Ogata M, Sata T, Chaudry IH (2010). Lidocaine enhances apoptosis and suppresses mitochondrial functions of human neutrophil in vitro. J Trauma.

[29] Hodgkin AL, Huxley AFA (1952). quantitative description of membrane current and its application to conduction and excitation in nerve. J Physiol.

[30] Besson P, Driffort V, Bon É, Gradek F, Chevalier S, Roger S (2015). How do voltage-gated sodium channels enhance migration and invasiveness in cancer cells?. Biochim Biophys Acta.

[31] Roger S, Gillet L, Le Guennec JY, Besson P (2015). Voltage-gated sodium channels and cancer: is excitability their primary role?. Front Pharmacol.

[32] Roger S, Potier M, Vandier C, Besson P, Le Guennec JY (2006). Voltage-gated sodium channels: new targets in cancer therapy?. Curr Pharm Des.

[33] Driffort V, Gillet L, Bon E, Marionneau-Lambot S, Oullier T, Joulin V, Collin C, Pagès JC, Jourdan ML, Chevalier S, Bougnoux P, Le Guennec JY , Besson P, Roger S (2014). Ranolazine inhibits NaV1.5-mediated breast cancer cell invasiveness and lung colonization. Mol Cancer.

[34] Nelson M, Yang M, Dowle AA, Thomas JR, Brackenbury WJ (2015). The sodium channel-blocking antiepileptic drug phenytoin inhibits breast tumour growth and metastasis. Mol Cancer.

[35] Yuan T, Li Z, Li X, Yu G, Wang N, Yang X (2014). Lidocaine attenuates lipopolysaccharide-induced inflammatory responses in microglia. J Surg Res.

[36] Brocco MC, Paulo DNS, Almeida CED, Carraretto AR, Cabral SA, Silveira AC, Gomez RS, Baptista JF (2012). A study of interleukin 6 (IL-6) and tumour necrosis factor-alpha (TNF-α) serum levels in rats subjected to faecal peritonitis and treated with intraperitoneal ropivacaine. Acta Cirurgica Brasileira.

[37] Piegeler T, Schläpfer M, Dull RO, Schwartz DE, Borgeat A, Minshall RD, Beck-Schimmer B (2015). Clinically relevant concentrations of lidocaine and ropivacaine inhibit TNFα-induced invasion of lung adenocarcinoma cells in vitro by blocking the activation of Akt and focal adhesion kinase. Br J Anaesth.

[38] Shankar S, Chen Q, Srivastava RK (2008). Inhibition of PI3K/AKT and MEK/ERK pathways act synergistically to enhance antiangiogenic effects of EGCG through activation of FOXO transcription factor. J Mol Signal.

[39] Qian J, Zou Y, Rahman JSM, Lu B, Massion PP. Synergy between phosphatidylinositol 3 kinase/Akt pathway and Bcl-xL in the control of apoptosis in adenocarcinoma cells of the lung. Mol 2009;8:101–9 10.1158/1535-7163.MCT-08-0973 PMC3110728 19139118

[40] Ortega MA, Fraile-Mart’ınez O, As’Unsolo A, Buj’an J, Garc’ıa-Honduvilla N, Coca S (2020). Signal transduction pathways in breast cancer: the important role of PI3K/Akt/mTOR. J Oncol.

[41] Royds J, Khan AH, Buggy DJ (2016). Update on existing ongoing prospective trials evaluating the effect of anaesthetic and analgesic techniques during primary Cancer surgery on Cancer recurrence or metastasis. Int Anesthesiol Clin.

[42] Burgering BMT, Medema RH (2003). Decision on life and death: FOXO forkhead transcription factors are in command when PKB/Akt is off duty. J Leukoc Biol.

[43] Chen Q, Ganapathy S, Singh KP, Shankar S, Srivastava RK (2010). Resveratrol Induces Growth Arrest and Apoptosis through Activation of FOXO Transcription Factors in Prostate Cancer Cells. PLoS ONE.

[44] Arnold A, Papanikolaou A (2005). (2005) Cyclin D1 in breast cancer pathogenesis. J Clin Oncol.

[45] Santarius T, Shipley J, Brewer D, Stratton MR, Cooper CS (2010). A census of amplified and overexpressed human cancer genes. Nat Rev Cancer.

[46] Datta SR, Brunet A, Greenberg ME (1999). Cellular survival: a play in three Akts. Genes DAev.

[47] Lirk P, Hollmann MW, Fleischer M, Weber NC, Feigl H (2014). Lidocaine, and ropivacaine, but not bupivacaine, demethylate deoxyribonucleic acid in breast cancer cells in vitro. Br J Anaesth.

[48] Li K, Yang J, Han X (2014). Lidocaine sensitizes the cytotoxicity of cisplatin in breast cancer cells via the up-regulation of RARβ2 and RASSF1A demethylation. Int J Mol Sci.

