# Supplementary figures and images for: Levobupivacaine inhibits proliferation and promotes apoptosis of breast cancer cells by suppressing the PI3K/Akt/mTOR signalling pathway
Source: BMC Res Notes. 2020 Aug 17;13:386. doi: 10.1186/s13104-020-05191-2 (PMC7430121; doi:10.1186/s13104-020-05191-2)

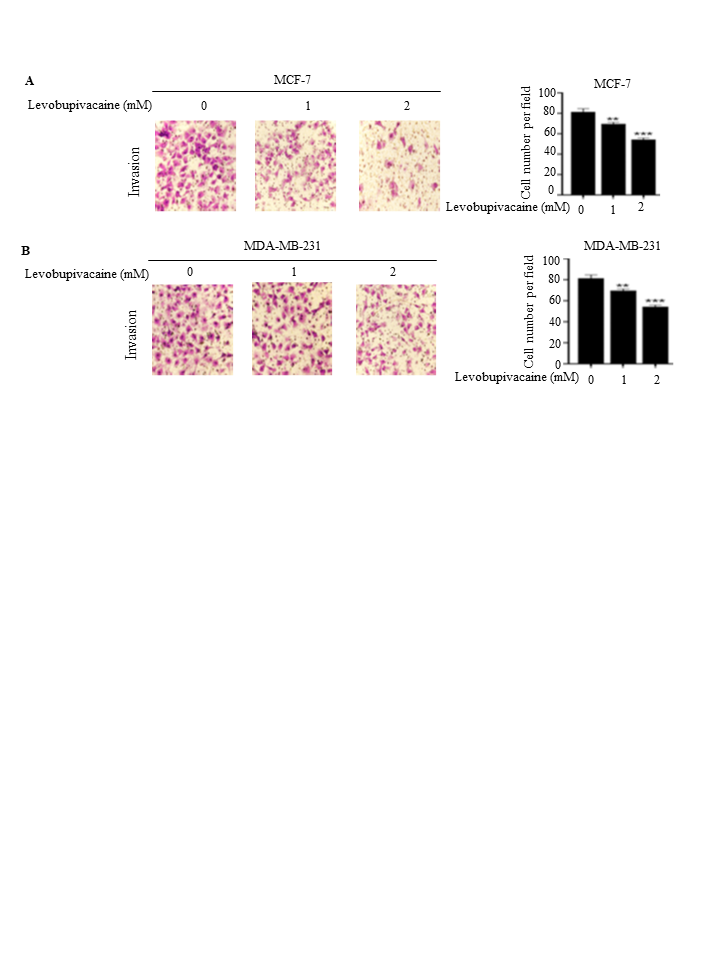

Supplement: Supplementary file 1 — Additional file 1: Figure S1 Levobupivacaine decreases breast cancer cell invasion. [file 13104_2020_5191_MOESM1_ESM.tif]

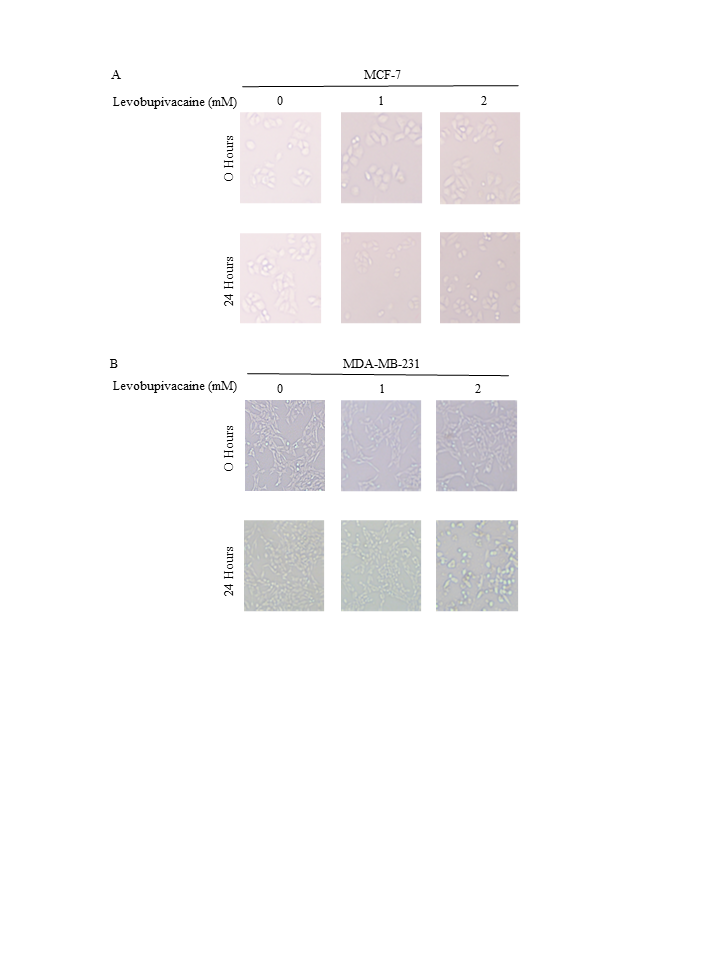

Supplement: Supplementary file 2 — Additional file 2: Figure S2 Effect of levobupivacaine on the morphology of MCF-7 and MDA-MB 231 cells. [file 13104_2020_5191_MOESM2_ESM.tif]
