# Supplementary material for: Levobupivacaine inhibits proliferation and promotes apoptosis of breast cancer cells by suppressing the PI3K/Akt/mTOR signalling pathway
Source: BMC Res Notes. 2020 Aug 17;13:386. doi: 10.1186/s13104-020-05191-2 (PMC7430121; doi:10.1186/s13104-020-05191-2)
Supplement: Supplementary file 3 — Additional file 3. Original gels/blots scan used in Fig. 1f, g; Fig. 2e, f and Fig. 3a, b for MCF-7 and MDA-MB-231 cells. [file 13104_2020_5191_MOESM3_ESM.docx]

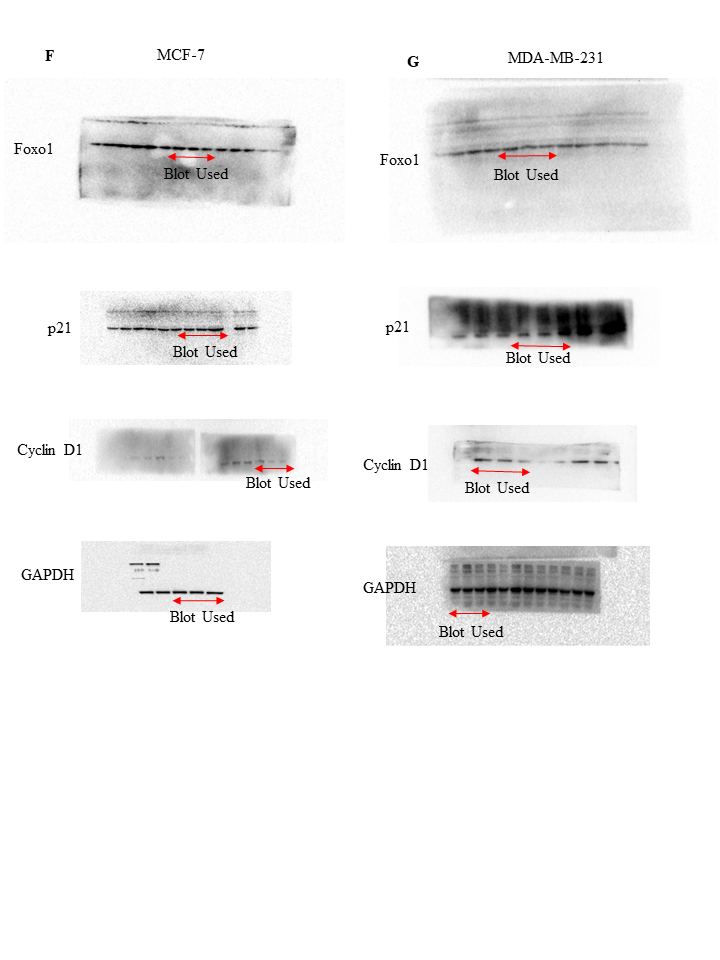


**Supplementary 3:** Full-length gels/plots images of western blots used in figure 1 F and G. MCF-7 and MDA-MB-231, gels/plots showing the expression of Foxo1, p21, Cyclin D1 and GAPDH following levobupivacaine treatment.


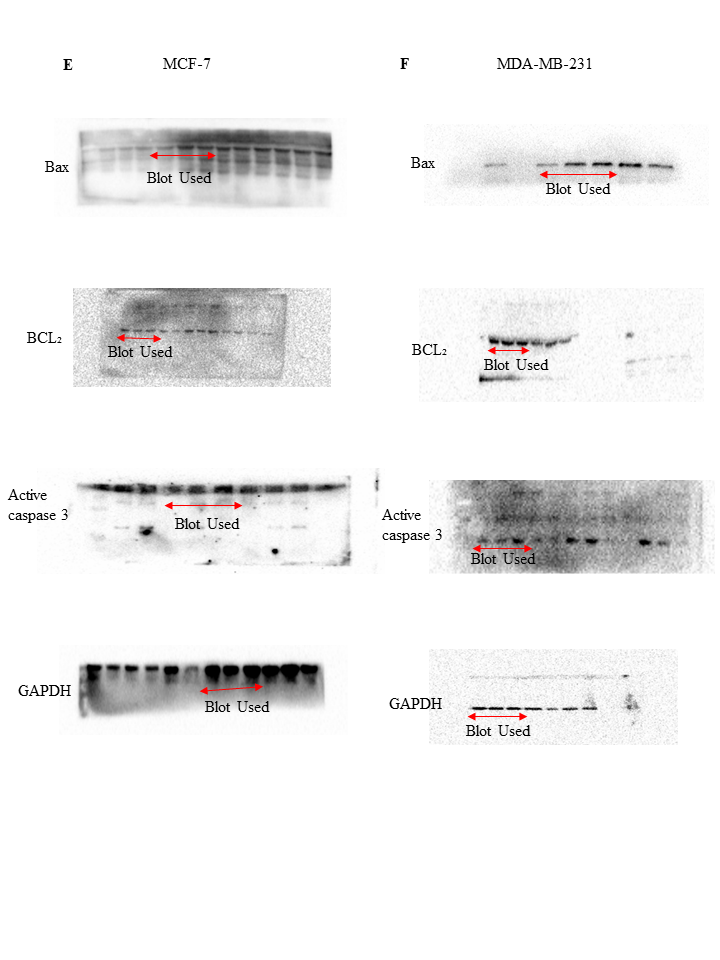


**Supplementary 3:** Full-length gels/plots images of western blots used in figure 2 E and F. MCF-7 and MDA-MB-231, gels/plots showing the expression of Bax, BCL_2_, Active

caspase 3 and GAPDH after treatment with levobupivacaine.


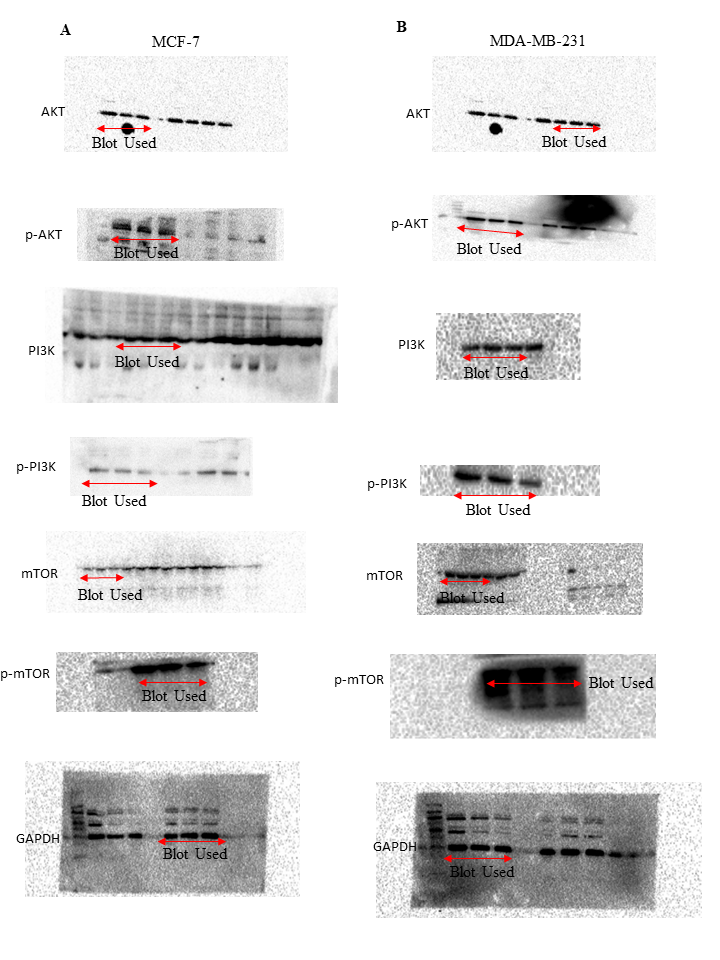


**Supplementary 3:** Full-length gels/plots images of western blots used in figure 3 A and B. MCF-7 and MDA-MB-231, gels/plots showing the expression of AKT, p-AKT, PI3K, p-PI3K, mTOR, p-mTOR and GAPDH following levobupivacaine treatment.
